# Polyunsaturated Fatty Acids of Both the Omega-3 and the Omega-6 Family Abrogate the Cytokine-Induced Upregulation of miR-29a-3p by Endothelial Cells

**DOI:** 10.3390/molecules25194466

**Published:** 2020-09-29

**Authors:** Daniel Maucher, Birte Schmidt, Kevin Kuhlmann, Julia Schumann

**Affiliations:** University Clinic and Outpatient Clinic for Anesthesiology and Operative Intensive Care, University Medicine Halle (Saale), 06112 Halle (Saale), Germany; daniel.maucher@uk-halle.de (D.M.); birteschmidt09@gmail.com (B.S.); kuhlmann-kevin@gmx.de (K.K.)

**Keywords:** endothelial dysfunction, miRNA, docosahexaenoic acid, arachidonic acid

## Abstract

Cellular processes fundamentally depend on protein expression control. At this, protein expression is regulated on the transcriptional and the post-transcriptional level. PUFAs are already known to affect gene transcription. The present study was conducted to answer the question whether PUFAs are also able to impact on the miRNA-mediated post-transcriptional fine-tuning of mRNA copy numbers. To this end, cellular miRNA profiles were screened by means of next-generation sequencing and NanoString analysis to compare PUFA-enriched to unsupplemented endothelial cells exposed to an inflammatory milieu. Validation took place by droplet digital PCR, allowing for an absolute quantification of RNA copy numbers. The analyses revealed that the stimulation-induced upregulation of miR-29a-3p is blocked by PUFA enrichment of endothelial cells. What is more, mRNA copy numbers of miR-29a-3p targets, namely the coagulation factors PAI-1, TF, and vWF, as well as the proinflammatory cytokines IL-1β, IL-6, and IL-8, were reduced in PUFA-enriched endothelial cells compared to unsupplemented cells, counteracting the stimulatory effect of an inflammatory environment. These data hint toward a new mechanism of action by which PUFAs modulate the functionality of endothelial cells. Apparently, the inflammation-modulating properties of PUFAs are also mediated at the post-transcriptional level.

## 1. Introduction

Polyunsaturated fatty acids (PUFAs) are components of the diet that are considered to have a significant impact on human health. Dietary fatty acids pass from the gastrointestinal tract into the bloodstream to be circulated throughout the body. This distribution mechanism implies that cells with constant blood contact, such as the endothelial cells lining the blood vessels, are particularly exposed to the influence of dietary PUFAs.

Endothelial cells play an important role in both acute and chronic inflammatory processes and can be directly activated by proinflammatory cytokines [1,2]. In the activated state, endothelial cells are characterized by the production of reactive oxygen and nitrogen intermediates as well as proinflammatory mediators, such as tumor necrosis factor-alpha (TNF-α), granulocyte-macrophage colony-stimulating factor (GM-CSF), monocyte chemoattractant protein 1 (MCP-1), and the interleukins IL-1β, IL-6, and IL-8 [1,2]. What is more, coagulation processes are facilitated and leukocyte adhesion and extravasation are promoted [1,2]. Accordingly, endothelial cells are regarded as part of a functional immune defense actively participating in the combat of invading pathogens. In order to achieve a physiologically meaningful reaction, however, the immunological activation of the endothelial cells must necessarily be temporary and spatially limited. A generalized activation of the endothelium represents a pathophysiological process and is the hallmark of numerous serious diseases, such as atherosclerosis or sepsis [3,4,5,6,7].

Investigations by our working group have already shown that the cytokine-driven activation of endothelial cells is modulated by unsaturated fatty acids [2]. In line with this, PUFAs are already used in clinical practice to accompany the therapy of inflammatory diseases. For example, the parenteral solutions employed in intensive care units for the treatment of sepsis patients are enriched with PUFAs.

PUFAs are known to act through a variety of mechanisms. They serve as precursors for the inflammation-mediating eicosanoids and resolvins, represent direct ligands of receptors associated to the immune response, and are known to be incorporated into plasma membrane phospholipids, thus modulating cellular signaling events [8]. According to the hitherto-described mechanisms of action, PUFAs thus either act by direct conversion into inflammatory mediators or by influencing the transcription rate of certain genes.

In fact, cellular processes fundamentally depend on gene expression control. However, not only the mRNA synthesis rate but also the mRNA stability and mRNA degradation rate affect the efficiency of protein biosynthesis. This post-transcriptional regulation is mediated by non-coding RNAs, such as microRNAs (miRNAs) [9]. miRNAs were first discovered in *Caenorhabditis elegans* in 1993, but their function remained neglected until 2001 [10]. The short and single-stranded RNAs are described to be expressed in a cell- and tissue-specific manner [11], thereby influencing the translation of their target mRNA(s) [12]. The miRNA-based post-transcriptional regulation is somewhat complex. On the one hand, a specific mRNA can be the target of various miRNAs [13,14]. On the other hand, one miRNA can recognize and influence different mRNAs [13,14].

So far, numerous miRNAs have been identified that influence the immune system’s defense mechanisms [15]. The importance of miRNAs in infectious processes is also reflected in the fact that changes in the miRNA profiles of immune cells have been detected in sepsis patients [15,16,17]. Remarkably, miRNAs have been shown to affect endothelial function and the vascular barrier [16,18,19,20]. The cytokine-mediated activation of endothelial cells is based on the initiation of signaling cascades whose mediators are known to be influenced by miRNAs [16,17,21,22,23]. Numerous studies also prove the modulating effect of miRNAs on the expression of the adhesion molecules e-selectin, ICAM-1, and VCAM-1, which are essential for the extravasation of leukocytes [17,18,21,22].

Feeding studies in rats have shown that the miRNA profile of different tissues and cell types, such as colon mucosa, pituicytes, peripheral blood mononuclear cells (PBMCs), adipocytes, and hepatocytes, differs depending on the PUFA content of the diet [24,25]. It is therefore reasonable to assume that PUFA supplementation also acts on the miRNA expression of endothelial cells. However, a scientific verification of this hypothesis has not yet been conducted, despite its potential medical importance.

Here, we present evidence that the miRNA expression profile of endothelial cells can be modulated by PUFAs. Both the omega-3 PUFA docosahexaenoic acid (DHA; C22:6n3) and the omega-6 PUFA arachidonic acid (AA; C20:4n6) were found to negatively affect the expression of the miRNA miR-29a-3p in an inflammatory setting. Remarkably, the target genes of miR-29a-3p include coagulation factors and cytokines, which play a crucial role in endothelial inflammatory processes. Indeed, PUFA enrichment of endothelial cells not only suppresses the cytokine-induced upregulation of miR-29a-3p but also counteracts the cytokines’ effect on the expression of those target genes. Our data therefore provide proof of a new mechanism of action by which PUFAs modulate the functionality of endothelial cells.

## 2. Results

### 2.1. Cytokine Treatment of Endothelial Cells Results in Distinct Alterations of the miRNA Profile

Two independent screening methods (next-generation sequencing and NanoString) were used to compare the miRNA profiles of unstimulated and cytokine-stimulated endothelial cells. In total, 14 miRNAs were identified, which were consistently indicated as altered expressed in both methods: miR-21-5p, miR-23b-3p, miR-26b-5p, miR-29a-3p, miR-29b-3p, miR-30d-5p, miR-30e-5p, miR-106b-5p, miR-146a-5p, miR-155-5p, miR-181a-5p, miR-181b-5p, miR-221-5p, and miR-342-3p.

**Figure 1:**
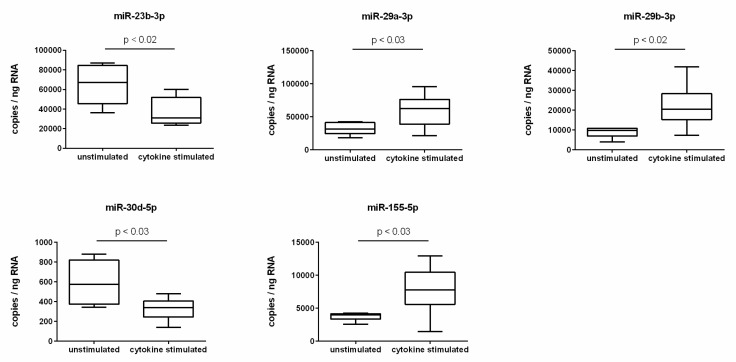
miRNA expression changes due to cytokine stimulation of endothelial cells.

To validate the cytokine-induced expression changes predicted by screening, absolute quantification using ddPCR was performed. The screening prognosis was confirmed for a total of five miRNAs. A significant reduction of expression in cytokine-treated endothelial cells was observed for the miRNAs miR-23b-3p and miR-30d-5p. For both miRNAs, the expression rate was decreased by a factor of 0.6 compared to unstimulated endothelial cells as a result of cytokine treatment (Figure 1). In addition, a cytokine-induced significant increase in expression was seen for the miRNAs miR-29a-3p, miR-29b-3p, and miR-155-5p. In cytokine-stimulated endothelial cells, the expression of miR-29a-3p was upregulated 1.9-fold, of miR-29b-3p 2.5-fold, and of miR-155-5p 2.1-fold (Figure 1).

Absolute quantification of miRNA expression was performed by ddPCR analysis using specific miRCURY LNA miRNA PCR assay primers (N = 6, n = 2). The endothelial cell line TIME was investigated after stimulation for 24 h with a cytokine mix consisting of IL-1β, TNF-α, and IFN-γ (each in a concentration of 5 ng/mL) in comparison to the unstimulated control. miRNA expression was measured as RNA molecules per ng of isolated total RNA. The displayed boxplots indicate the median of the determined miRNA expression values, the lower and upper quantiles, and the two extreme values.

### 2.2. PUFA-Enriched Endothelial Cells Show No Upregulation of miR-29a-3p after Cytokine Stimulation

**Figure 2:**
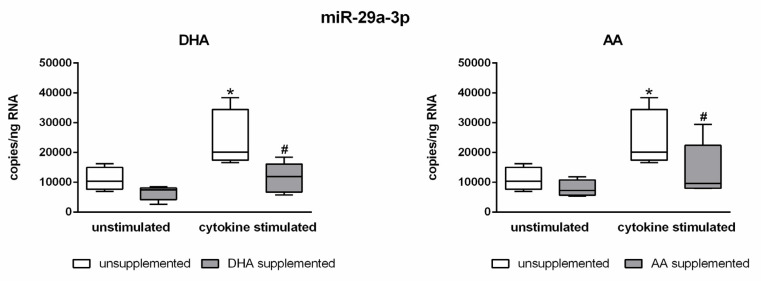
Abrogation of the cytokine effect on the expression of miR-29a-3p by DHA and AA.

Next, a potential interference of PUFAs with the observed cytokine-induced changes in miRNA expression of endothelial cells was investigated. Indeed, a clear PUFA effect was found for the miRNA miR-29a-3p. The enrichment of the endothelial cells with the omega-3 fatty acid DHA or the omega-6 fatty acid AA caused an almost complete suppression of the cytokine-mediated upregulation of miR-29a-3p. That is, in PUFA-enriched cells, the expression level of miR-29a-3p was constant regardless of the presence or absence of the proinflammatory cytokines IL-1β, TNF-α, and IFN-γ (Figure 2). It is remarkable that PUFA supplementation itself did not have an effect on miRNA expression. The expression range of miR-29a-3p was comparable between unsupplemented and PUFA-enriched endothelial cells under control conditions. A PUFA effect only became apparent in a proinflammatory milieu (Figure 2).

Absolute quantification of miRNA expression was performed by ddPCR analysis (n = 5, n = 3). The endothelial cell line TIME was investigated after supplementation with either docosahexaenoic acid (DHA) or arachidonic acid (AA) for 144 h. Stimulation of cells was performed in the last 24 h of fatty acid supplementation by means of a cytokine mix consisting of IL-1β, TNF-α, and IFN-γ (each in a concentration of 5 ng/mL). Unsupplemented and/or unstimulated cells served as a control. miRNA expression was measured as RNA molecules per ng of isolated total RNA. The displayed boxplots indicate the median of the determined miRNA expression values, the lower and upper quantiles, and the two extreme values. * indicates significant differences between unsupplemented/unstimulated and unsupplemented/cytokine-stimulated endothelial cells. # indicates significant differences between unsupplemented/cytokine-stimulated and supplemented/cytokine-stimulated endothelial cells.

### 2.3. Central Mediators of Endothelial Dysfunction are Targeted by miR-29a-3p

Using the in silico database miRWalk2.0, the target genes of miR-29a-3p were identified. The analysis showed that central mediators of endothelial dysfunction are among the predicted target genes. On the one hand, the group of coagulation factors came into focus. Putative target genes of miR-29a-3p from this group are SERPINE1 (encoding the plasminogen activator inhibitor-1 (PAI-1), an inhibitor of fibrinolysis), F3 (encoding the tissue factor (TF), a member of the coagulation cascade), and VWF (encoding the von Willebrand factor (vWF), an initiator of platelet adhesion). On the other hand, the group of cytokines was spotlighted. Predicted target genes of miR-29a-3p from this group are IL1b (encoding the proinflammatory interleukin 1β (IL-1β), IL6 (encoding the proinflammatory interleukin 6 (IL-6), and CXCL8 (encoding the chemotactic interleukin 8 (IL-8)).

### 2.4. Target Genes of miR-29a-3p Are Subject to the Modulating Effect of PUFAs

**Figure 3:**
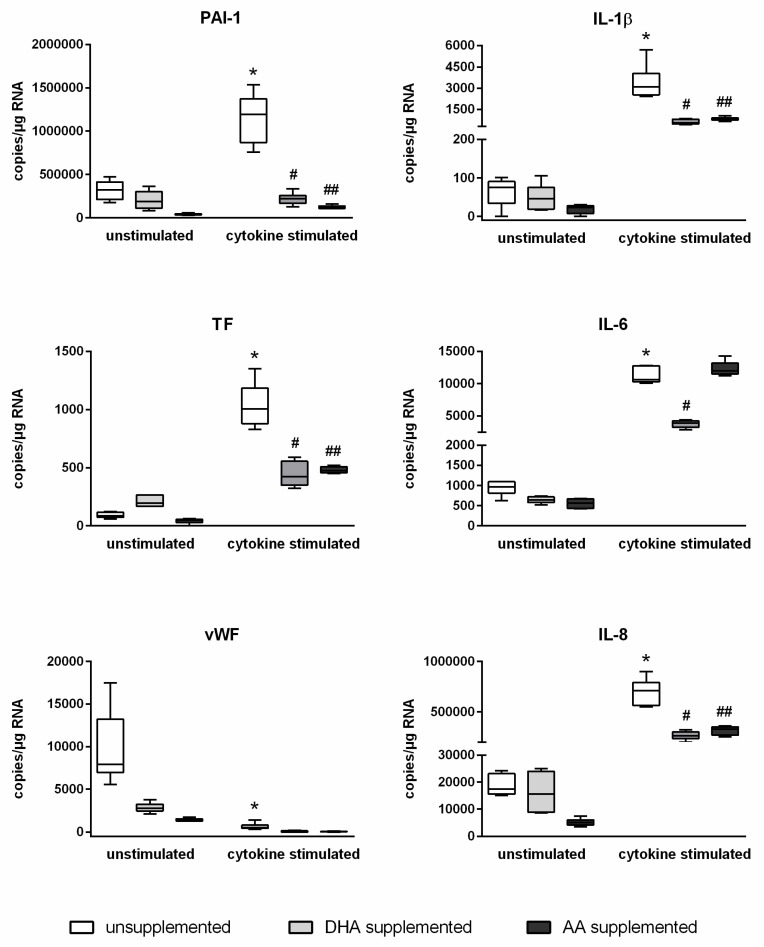
Abrogation of the cytokine effect on the expression of PAI-1, TF, vWF, IL-1β, IL-6, and IL-8 by DHA and AA.

An inflammatory milieu distinctly affects the gene expression of endothelial cells. This also applies to the identified target genes of miR-29a-3p. Stimulated endothelial cells showed markedly increased mRNA expression levels of PAI-1, TF, IL-1β, IL-6, and IL-8 and a significantly decreased mRNA expression level of vWF (Figure 3). Enrichment of endothelial cells with PUFAs revealed a changed scenario, whereby PUFA effects were observed for all investigated target genes of miR-29a-3p. The stimulation-induced upregulation of mRNA copy numbers of PAI-1, TF, IL-1β, IL-6, and IL-8 was evidently diminished in fatty acid-supplemented cells (Figure 3). Additionally, the stimulation-induced downregulation of mRNA copy numbers of vWF was found by the trend to be further enhanced in PUFA-enriched endothelial cells (Figure 3). PUFA enrichment of endothelial cells thus affects both the expression of the miR-29a-3p itself and the expression of the target genes of this miRNA. This suggests a causal link. The data indicate that PUFAs influence the copy number of a specific mRNA available for protein synthesis by influencing endothelial miRNA expression.

Absolute quantification of mRNA expression was performed by ddPCR analysis (n = 6, n = 2). The endothelial cell line TIME was investigated after supplementation with either docosahexaenoic acid (DHA) or arachidonic acid (AA) for 144 h. Stimulation of cells was performed in the last 24 h of fatty acid supplementation by means of a cytokine mix consisting of IL-1β, TNF-α, and IFN-γ (each in a concentration of 5 ng/mL). Unsupplemented and/or unstimulated cells served as a control. mRNA expression was measured as RNA molecules per µg of isolated total RNA. The displayed boxplots indicate the median of the determined miRNA expression values, the lower and upper quantiles, and the two extreme values. * indicates significant differences between unsupplemented/unstimulated and unsupplemented/cytokine-stimulated endothelial cells. # indicates significant differences between unsupplemented/cytokine-stimulated and DHA-supplemented/cytokine-stimulated endothelial cells. ## indicates significant differences between unsupplemented/cytokine-stimulated and AA-supplemented/cytokine-stimulated endothelial cells.

## 3. Discussion

The inflammation-modulating properties of PUFAs have long been the subject of research. It is known that PUFAs achieve their effects through a number of different mechanisms. PUFAs act as direct ligands of the receptors peroxisome proliferator-activated receptor gamma (PPARγ) and the G-protein coupled receptor 120 (GPR120), thus triggering predominantly anti-inflammatory signaling cascades [8]. Furthermore, fatty acids are essential components of phospholipids and thus of the cellular plasma membrane. When available, unsaturated fatty acids are incorporated into the membrane, which changes the chemical and physical properties of the membrane domains [8,26,27]. This also affects the protein–protein interactions of membrane receptors. Previous research of our working group has shown that the lipopolysaccharide (LPS)-induced interaction of the toll-like receptor 4 (TLR4) with its co-receptor CD14 is disrupted upon membrane enrichment with PUFAs, resulting in an inhibition of the signaling cascade [28] and a blockade of the LPS-mediated activation of the transcription factor nuclear factor kappa B (NFκB), which is central to inflammatory processes [29]. Furthermore, PUFAs are the precursors of a number of inflammatory mediators. On the one hand, the group of eicosanoids needs to be pointed out. Eicosanoids mediate proinflammatory effects, whereby the effectiveness of omega-6 derivatives proves to be more potent than that of omega-3 derivatives [8]. On the other hand, resolvins, which perform important tasks in inflammation resolution, are also derived from PUFAs [8].

In the present study, DHA and AA, two central representatives of omega-3 and omega-6 fatty acids, were specifically selected to investigate a possible effect of PUFAs of both subclasses on the post-transcriptional regulation of gene expression. This was done against the background of an ongoing discussion of the role of omega-3 and omega-6 fatty acids in inflammatory processes. It is generally assumed that PUFAs of the omega-3 family, such as DHA or eicosapentaenoic acid (EPA; C20:5n3), are of crucial importance for inflammatory resolution [8]. However, the impact of omega-6 fatty acids appears less clear. On the one hand, AA serves as a precursor of potent prostanoids that promote inflammation [30]. Accordingly, many nonsteroidal anti-inflammatory drugs (NSAID), such as acetylsalicylic acid, act as enzyme inhibitors that prevent the synthesis of AA-derived prostanoids. On the other hand, some AA-derived resolvins promote the resolution of an inflammatory process [30]. Consequently, both proinflammatory and anti-inflammatory effects have been observed in the supplementation of healthy adults with AA [30]. A previous study of the working group on endothelial cells, which used identical cultivation, supplementation, and stimulation conditions as the present work, can serve as an example. Here, an inhibitory effect of AA on the cytokine-induced upregulation of the adhesion molecule VCAM-1 was found, an effect that had previously only been described for DHA and EPA [2]. Additionally, the effects of DHA and AA described in the present study also point consistently in the anti-inflammatory direction for both fatty acid families.

The description of miRNAs opens up the possibility of a further mechanism of action of PUFAs. miRNAs are of crucial importance for the stability of cellular mRNAs. In the presence of at least partial complementarity, miRNAs and mRNAs interact with each other, modulating the rate of mRNA degradation. Both inhibitory and promoting effects are described [31,32,33]. It is clear that the type and quantity of miRNAs present in a cell affect the number of mRNA copies available for translation. Thus, by modulating miRNA expression, it is possible to influence protein expression and consequently the biological functionality of a cell.

It is known that the presence of an inflammatory milieu impacts on the miRNA profile of a cell [34]. This was also confirmed in our study. The stimulation of endothelial cells with the cytokines IL-1β, TNF-α, and IFN-γ was accompanied by a downregulation of the miRNAs miR-23b-3p and miR-30d-5p and an upregulation of the miRNAs miR-29a-3p, miR-29b-3p, and miR-155-5p. We were now interested in how PUFA enrichment of endothelial cells affects the process of miRNA expression. Indeed, we found that the stimulation-induced upregulation of miR-29a-3p in PUFA-enriched endothelial cells was completely blocked.

Cytokine stimulation of cells induces signaling cascades that ultimately culminate in the activation of transcription factors. Considering the cytokine mix of IL-1β, TNF-α, and IFN-γ used in our experiments, among others, an upregulation of the transcription factors CCAAT/enhancer-binding protein α and β (C/EBPα, C/EBPβ), NFκB subunit p65, activator protein 1 (AP-1), and signal transducer and activator of transcription 1 (STAT1) can be assumed (KEGG Pathway Database; [35]). Each of these transcription factors is capable of influencing the expression of miR-29a-3p, since specific binding sites have been shown to be part of the promoter and enhancer elements of the gene coding for miR-29a-3p (GeneCards; [36]). At this point, it is important to realize that not only cytokines but also PUFAs interfere with the expression and activation of the stated transcription factors. Feeding studies in rats demonstrate an inhibitory effect of PUFA-enriched nutrition on the expression of C/EBPα [37]. In core extracts of primary vascular smooth muscle cells, reduced protein levels of C/EBPβ were detected after omega-3-PUFA supplementation, which correlated with a reduction of IL-1β-induced C/EBP binding activity in an electrophoretic mobility shift assay (EMSA) [38]. Similar effects were also found for p65 [38]. This corresponds to previous data from our group that show PUFA-mediated inhibition of NFκB activity utilizing a luciferase reporter gene assay [29]. In addition, an inhibitory effect on AP-1 activity was found for DHA, which was due to a suppression of stimulation-induced DNA binding by AP-1 [39]. There are also reports of blocking the IFN-γ-mediated phosphorylation of STAT1 by PUFA of both the omega-3 and omega-6 family (DHA, EPA, AA) [40,41]. In summary, a conclusive mechanistic approach is the result. Our data together with the findings from the scientific literature suggest that the observed inhibitory effect of PUFAs on the expression of miR-29a-3p is due to the interference of PUFA with the transcription factors C/EBP, p65, AP-1, and STAT1.

The analysis of the target genes of miR-29a-3p revealed that essential mediators of an endothelial inflammatory response, namely the coagulation factors PAI-1, TF, and vWF, as well as the cytokines IL-1β, IL-6, and IL-8, may be subject to the influence of miR-29a-3p. Therefore, it is reasonable to assume that PUFAs, by inhibiting the stimulation-mediated upregulation of miR-29a-3p, make an impact on the post-transcriptional regulation of these central inflammation-associated factors. In fact, under inflammatory conditions, the detectable mRNA copy numbers of PAI-1, TF, vWF, IL-1β, IL-6, and IL-8 in PUFA-supplemented cells were significantly altered compared to unsupplemented cells. In each case, there was a reduction in mRNA copies, which in the example of PAI-1 completely cancelled out the stimulation effect.

Our study thus provides first indications of a new mechanism of action of PUFAs. We were able to show that the enrichment of endothelial cells with PUFAs inhibits the upregulation of a specific miRNA in the inflammatory milieu, which had a direct effect on the copy numbers of the target mRNAs of this miRNA available for translation. This is the first evidence that the inflammation-modulating properties of PUFAs are also mediated post-transcriptionally. The elegance of the mechanism as well as the universality of miRNA-mediated modulation of protein expression suggests that the described principle is transferable to other inflammation-associated cell types. The proposed mechanism of action is therefore not only important for the understanding of the PUFA-mediated modulation of endothelial dysfunction but also for the overall understanding of the immunomodulatory effects of PUFAs.

## 4. Materials and Methods

### 4.1. Culturing and Stimulation of Cells

The human cell line TIME (ATCC^®^ number CRL-4025) was used as a model of microvascular endothelial cells. Culturing of TIME was performed at 37 °C and 5% CO_2_ in a humidified atmosphere using basal microvascular endothelial growth medium (Provitro, Berlin, Germany) containing 5 ng/mL VEGF, 5 ng/mL EGF, 5 ng/mL FGF, 15 ng/mL IGF-1, 10 mM L-glutamine, 0.75 U/mL heparin sulfate, 1 µg/mL hydrocortisone hemisuccinate, 50 µg/mL ascorbic acid, 5% *v*/*v* FCS, and 12.5 µg/mL blasticidin. PUFA supplementation was carried out for 144 h. This period of supplementation was previously proven to result in a membrane fatty acid steady state [2]. The PUFAs docosahexaenoic acid (DHA; C22:6n3) or arachidonic acid (AA, C20:4n6) (both Biotrend, Köln, Germany) were included in the culture medium at a final concentration of 15 µmol/L using ethanol as a vehicle (0.1% *v*/*v* final ethanol concentration). Stimulation of cells was performed in the last 24 h of fatty acid supplementation by addition of the cytokines IL-1β, TNF-α, and IFN-γ (all PeproTech, Hamburg, Germany), each in a concentration of 5 ng/mL.

### 4.2. RNA Isolation

Total RNA extraction was performed using a standard extraction protocol based on TRIzol (Thermo Fisher Scientific, Dreieich, Germany) according to the manufacturer′s instructions. The concentration and quality of RNA gained were analyzed by means of the NanoDrop spectrophotometer (Thermo Fisher Scientific, Dreieich, Germany) as well as the Agilent Bioanalyzer (Agilent Technologies, Waldbronn, Germany). An absorbance quotient A260/280 > 1.8 was considered appropriate for following procedures.

### 4.3. miRNA Expression Screening

Screening for differentially expressed miRNAs was performed by means of two independent procedures: Next-generation sequencing (NGS) and the NanoString technique. Three biological replicates were analyzed in every test group. miRNAs, which were detected with a high abundance (≥100 reads) in both methods, were examined for cytokine-induced expression changes. Only those miRNAs were considered that were consistently indicated as having changed expression in both methods and that had a high effect strength (Cohen′s d ≥ 0.8/≤−0.8). The deep sequencing datasets generated and analyzed during the current study are available in the Gene Expression Omnibus (GEO) repository, accession number GSE132361.

NGS analysis was carried out in the Core Unit DNA, Leipzig University by means of an Illumina HiScanSQ (Illumina Inc., San Diego, CA, USA). Samples were processed using the TruSeq Small RNA Prep kit v2 (Illumina Inc., San Diego, CA, USA) following the manufacturer’s standard protocol. Size restriction (140–165 bp), purification, and quantification of barcoded libraries were performed using the Library quantification kit-Illumina/Universal (KAPA Biosystems, Woburn, MA, USA). For cluster generation, up to 10 libraries per lane were factored using an Illumina cBot. Then, 50 bp sequencing was performed using an Illumina HighScanSQ sequencer based on version 3 chemistry and flowcell following the manufacturer’s standard protocol. For deep sequencing data analysis, the adapter sequences were removed from raw sequences by means of Cutadapt software. Only 15–27-base-long sequences were analyzed. These reads were aligned to the human genome as well as mature sequences of miRBase v21 using the bowtie aligner. For data compression to bam format, Samtools were used. Mapped reads count was determined using the R/Bioconductor programming environment by application of the ShortRead library. An error ratio of 1 nt per mature miRNA sequence was accepted. Normalization of data was performed by independent application of the RPM and the TMM algorithm.

NanoString analysis was carried out at the Institute of Human Genetics, Martin Luther University Halle-Wittenberg by means of the nCounter FLEX Analysis System (NanoString Technologies, Hamburg, Germany) using the nCounter human v3 miRNA Expression Assay according to the manufacturer’s instructions. Normalization of the generated data was performed using the nSolverTM Version 3.0 software. First, the background noise was subtracted with the help of the miRNA assay negative controls. This was followed by a technical normalization using the miRNA assay positive controls. Finally, to compensate for potential differences in the amount of total RNA used between the samples, a biological normalization was performed based on the comparison of the 100 most strongly expressed miRNAs and the so-called positive ligation controls.

### 4.4. cDNA Synthesis

For miRNA analysis, complementary DNA (cDNA) was synthesized from all RNA samples by means of the miRCURY LNA RT Kit (QIAGEN, Hilden, Germany) according to standard protocol. As a positive control, the spike-in RNA UniSp6 was added to the samples before synthesis.

For mRNA analysis, cDNA was synthesized from all RNA samples by means of the qScript cDNA SuperMix (Quantabio, Berverly, MA, USA) according to standard protocol. As a positive control, cDNA was also synthesized from human heart aorta total RNA (Takara Bio Europe SAS, Saint-Germain-en-Laye, France).

### 4.5. Droplet Digital PCR (ddPCR)

miRNA and mRNA copy counts were determined by means of the housekeeper-independent Droplet Digital PCR technology (BioRad, Munich, Germany) following the manufacturer’s standard protocols and using ddPCR EvaGreen Supermix (Bio-Rad, Munich, Germany). For miRNA analysis, appropriate miRCURY LNA miRNA PCR assay primers (QIAGEN, Hilden, Germany) were applied. These primers are designed to be used consistently with a given amplification protocol (40 cycles: 30 s at 95 °C, 60 s at 58 °C, ramp rate 1.6 °C per second). For mRNA analysis, primers were purchased from Eurofins Genomics Germany (Ebersberg, Germany) and used according to the specified amplification conditions listed in Table 1. The ddPCR reaction was performed in a T100 Thermal Cycler (Bio-Rad, Munich, Germany). Measurement of positive droplets per µL of sample was performed on a QX200 ddPCR Droplet Reader (Bio-Rad, Munich, Germany). Based on the droplet count and according to Poisson distribution, the absolute nucleic acid copy count was calculated. Data output was converted into nucleic acid copy count per ng of RNA. ddPCR reaction was performed in duplicates to triplicates of five to six biological replicates in every test group.

### 4.6. In Silico Analysis

The miRWalk2.0 database (http://zmf.umm.uni-heidelberg.de/apps/zmf/mirwalk2/index.html) was used to identify the target genes of differentially expressed miRNAs. Validated target genes as well as target genes predicted on the basis of sequence analogies were determined. The predictions of all databases available at miRWalk2.0 were taken into account. Based on the databases miRWalk2.0 and miRBase (http://www.mirbase.org/), miRNA-specific information regarding gene family, transcription, genloki, and presence in gene clusters were also investigated.

### 4.7. Statistical Analysis

If not stated otherwise, data are shown as median ± interquartile range (IQR). To identify significant differences, an unpaired *t* test was used in two group comparisons and a single factor analysis of variance (ANOVA) with post-hoc test according to Bonferroni–Šídák was used in multiple group comparisons. The statistical analysis was carried out by means of the GraphPad Prism 6 software (GaphPad Software, La Jolla, CA, USA). A *p* value < 0.05 was assumed as an indicator of significant differences.

## Figures and Tables

**Table 1:** Target, primer sequence, annealing temperature (X), and elongation time (Y) used for mRNA analysis. Cycling conditions were as follows: initial denaturation for 3 min at 95 °C, followed by 44 cycles of 10 s denaturation at 95 °C, 10 s annealing at X °C, and extension at 72 °C for Y second. Abbreviation: IL-1β = interleukin-1beta, IL-6 = interleukin-6, IL-8 = interleukin-8, PAI-1 = plasminogen activator inhibitor-1, TF = tissue factor, vWF = von Willebrand factor.

Target	Primer Sequence (5′→3′)	Annealing Temperature [°C]	Extension Time [s]
IL-1β	ACGCTCCGGGACTCACAGCA TGAGGCCCAAGGCCACAGGT	66	20
IL-6	AAGCCAGAGCTGTGCAGATG CTGGCATTTGTGGTTGGGTC	56	10
IL-8	CCTGATTTCTGCAGCTCTGTG CCAGACAGAGCTCTCTTCCAT	56	20
PAI-1	CAGACCAAGAGCCTCTCC ATCACTTGGCCCATGAAAAG	54	20
TF	CACAGAGTGTGACCTCACCG ATTGTTGGCTGTCCGAGGTT	60	20
vWF	GGCAATTCCTTCCTCCACAAAC CAGTTGACCCGATGACTCTTCA	61	20
